# Topical Delivery Systems Effectively Transport Analgesics to Areas of Localized Pain via Direct Diffusion

**DOI:** 10.3390/pharmaceutics15112563

**Published:** 2023-10-31

**Authors:** Thomas Birngruber, Kip Vought, Simon Schwingenschuh, Peter Reisenegger, Howard Maibach, Dmitri Lissin

**Affiliations:** 1 HEALTH—Institute for Biomedical Research and Technologies, Joanneum Research Forschungsgesellschaft mbH, 8010 Graz, Austria; simon.schwingenschuh@joanneum.at (S.S.); peter.reisenegger@joanneum.at (P.R.); 2 Scilex Holding Company, Palo Alto, CA 94303, USA; kvought@scilexpharma.com; 3 Department of Dermatology, University of California San Francisco, San Francisco, CA 94143, USA; howard.maibach@ucsf.edu

**Keywords:** topical delivery system, analgesics, diclofenac, open flow microperfusion, direct diffusive transport, lidocaine patch, pharmacokinetics, musculoskeletal pain, porcine model, deep percutaneous penetration

## Abstract

Topical delivery systems (TDSs) enable the direct transport of analgesics into areas of localized pain and thus minimize the side effects of administration routes that rely on systemic drug distribution. For musculoskeletal pain, clinicians frequently prescribe topical products containing lidocaine or diclofenac. This study assessed whether drug delivery from a TDS into muscle tissue occurs mainly via direct diffusion or systemic transport. An investigational TDS containing 108 mg lidocaine (SP-103, 5.4% lidocaine), a commercially available TDS containing 36 mg lidocaine (ZTlido^®^, 1.8% lidocaine), and a topical pain relief gel (Pennsaid^®^, 2% diclofenac) were tested. Using open flow microperfusion (OFM), interstitial fluid from the dermis, subcutaneous adipose tissue (SAT), and muscle was continuously sampled to assess drug penetration in all tissue layers. Ex vivo and in vivo experiments showed a higher diffusive transport of lidocaine compared to diclofenac. The data showed a clear contribution of diffusive transport to lidocaine concentration, with SP-103 5.4% resulting in a significantly higher lidocaine concentration in muscle tissue than commercially available ZTlido^®^ (*p* = 0.008). These results indicate that SP-103 5.4% is highly effective in delivering lidocaine into muscle tissue in areas of localized pain for the treatment of musculoskeletal pain disorders (e.g., lower back pain).

## 1. Introduction

The most common ways to introduce drugs into the body are oral, intravenous, subcutaneous, or topical application. Topical delivery systems (TDSs) enable slow and continuous drug delivery for hours or even days, especially when adhesive patch systems are used [1]. Adhesive TDSs are particularly useful for drugs with a short half-life in blood and can have an additional benefit when applied near the target tissue; diffusive penetration from the patch directly into the underlying tissue layers enables effective drug delivery without relying on systemic drug transport. Especially for pain treatment, topical application of analgesics has become increasingly popular [2,3,4]. The systemic distribution of analgesics can lead to treatment-limiting adverse effects and sometimes even physical drug dependence [5,6,7,8]. Topical pain treatment has multiple advantages over systemically administered therapies such as site-specific drug delivery, pain relief at lower daily doses, and avoidance of drug interactions and first-pass metabolism [2,4,9]. Absorption and subsequent penetration of a drug into deeper tissues (e.g., muscle) is a complex process limited by various factors [10]. The drug’s molecular size, lipophilicity, and protein/tissue binding capacities affect the diffusion through the stratum corneum, epidermis, dermis, and subcutaneous adipose tissue (SAT) into the muscle [11,12]. Capillaries within these tissues provide the route to systemic drug delivery [13], but it is not yet fully understood to what extent diffusion, systemic uptake and redistribution, or a combination of both affect a drug’s efficacy in treating local pain [14]. For the treatment of musculoskeletal and neuropathic pain (e.g., lower back pain), clinicians frequently prescribe adhesive topical delivery systems (TDSs), also referred to as “patches” or “plasters”, containing lidocaine, a local anesthetic [2,3,15]. Such TDSs are expected to directly deliver the drug into the target tissue and are thus effective in treating local pain, especially in superficial muscle tissue [16,17]. It is often incorrectly assumed that the higher the analgesic concentration in these TDSs, the more effective they are in treating pain. However, the indicated percentage of the drug on the product label is merely the drug-to-adhesive ratio which cannot be directly translated into the amount of the drug that is actually delivered to the target tissue [11]. It is yet unknown what amount of the drug that is released from a TDS is effectively transported into muscle tissue via diffusion or systemic distribution, which also affects the optimal placement of an adhesive TDS.

The aim of our studywas to assess the contribution of the direct diffusive pathway and the systemic delivery pathway for transporting a drug into muscle tissue. First, we tested an investigational lidocaine-containing TDS (SP-103, 5.4% lidocaine) and a commercially available diclofenac-containing gel (Pennsaid^®^ 2%) ex vivo by using freshly explanted porcine tissue, a setup that excludes any influence of blood circulation. Second, to assess the contribution of systemic drug delivery, we tested Pennsaid^®^ 2%, SP-103 5.4%, and a commercially available lidocaine-containing TDS (ZTlido^®^, 1.8% lidocaine) in vivo in anesthetized pigs. In both experiments (ex vivo, in vivo), open flow microperfusion (OFM) [18] was used to continuously sample interstitial fluid (ISF) from dermal tissue, SAT, and muscle tissue to assess the temporal and spatial distribution of lidocaine and diclofenac delivered from the tested products.

## 2. Materials and Methods

### 2.1. Animals

All animal care and experimental procedures were approved by the Animal Testing Commission of the Federal Government of the Austrian Federal Ministry of Education, Science and Research (#2020-0.547.803). Domestic pigs (11.8 ± 1.0 weeks old, 38.3 ± 5.3 kg) were locally purchased and housed at the Division of Biomedical Research at the Medical University of Graz, Austria, under standard husbandry conditions.

### 2.2. Chemicals and Equipment

For the experiments, the following reagents were used: human serum albumin (Kedrion Biopharma, Vienna, Austria), ELO-MEL isotone, propofol, electrolyte solution, potassium chloride (Fresenius Kabi AG, Graz, Austria), midazolam, fentanyl (hameln pharma GmbH, Hameln, Germany), azaperone (Stresnil 40 mg/mL, Elanco, Vienna, Austria), ketamine (Ketasol, Ogris Pharma, Wels, Austria), sevoflurane (Sevorane, Abbott, Vienna, Austria), SP-103 5.4%, ZTlido^®^ 1.8% (Scilex Pharmaceuticals Inc., Palo Alto, CA, USA), Pennsaid^®^ 2 g/100 g gel (Gebro Pharma AG, Liestal, Switzerland), MilliQ water (Merck Millipore, Darmstadt, Germany), acetonitrile (Honeywell International Inc., Charlotte, NC, USA).

For ultrasound imaging, we used a LOGIQ e R6 (GE Healthcare, Amersham, UK) and ultrasound gel (Aquasonic 100, Parker Laboratories Inc., Fairfield, NJ, USA), and for temperature and humidity testing, a data logger (Testo 175 H1, Testo, Vienna, Austria) was used.

For HPLC-MS/MS, an Agilent UHPLC1290 and a G6495B triple quadrupole mass spectrometer (Agilent Technologies, Santa Clara, CA, USA) with a Waters Acquity UPLC BEH C8 column (Waters Corp., Milford, MA, USA) were used.

### 2.3. Tested Topical Delivery Systems (TDSs)

Two lidocaine-containing adhesive TDSs of different strengths and one diclofenac-containing topical gel were investigated (Table 1). In the ex vivo experiment, drug penetration of an investigational TDS (SP-103 5.4%) containing 108 mg lidocaine and a topical gel, Pennsaid^®^ 2% (2 g diclofenac/100 g gel), was investigated. Pennsaid^®^ 2% was selected as a test item because it is commonly used to treat musculoskeletal pain. Various studies have shown that topical diclofenac can diffuse into deeper tissues; however, a diffusion sufficient to reach the muscle has never been shown [19,20].

In the in vivo experiment, the commercially available lower-strength adhesive TDS ZTlido^®^ 1.8% was directly compared to SP-103 5.4%. SP-103 5.4% and ZTlido^®^ 1.8% are both drug-in-adhesive systems that share the same non-aqueous adhesive formulations and materials of composition, but SP-103 5.4% contains a 3-fold increased drug load relative to ZTlido^®^ 1.8%. Pennsaid^®^ 2% was used as a negative control for in vivo lidocaine delivery.

### 2.4. Open Flow Microperfusion (OFM)

**Figure 1:**
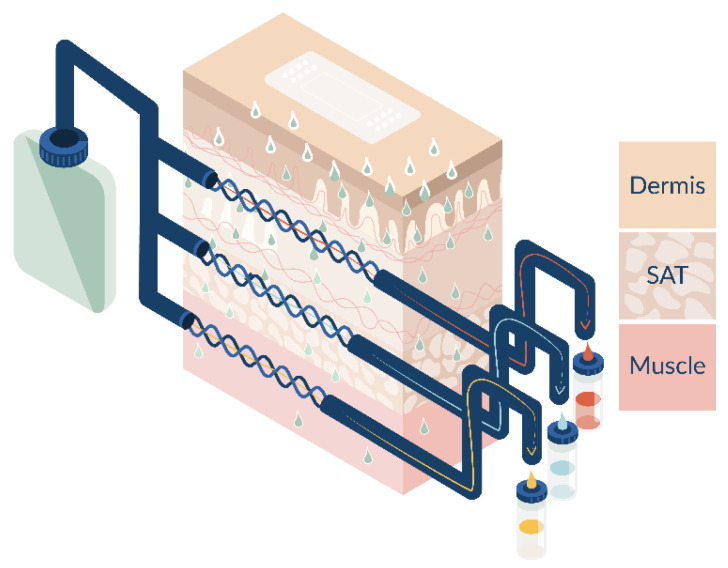
Schematic of OFM probe placement to test drug penetration in three tissue layers: dermis, subcutaneous adipose tissue (SAT), and muscle.

OFM is a minimally invasive probe-based sampling technology that allows continuous sampling of ISF in different target tissues. Linear OFM probes are made from polyether ether ketone (PEEK) with a 0.55 mm outer diameter and feature a 15 mm long exchange area with an open mesh design. OFM probes were implanted at six application sites either in tissue explants or on a pig’s back. At each application site, two OFM probes were inserted into the dermal layer of the skin, two in the SAT, and two in the muscle tissue (Figure 1). After insertion, OFM probes were first flushed with OFM perfusate (ELO-MEL isotone) and 2% human serum albumin for 10 min at a flow rate of 10 µL/min, which was then reduced to the standard flow rate of 0.5 µL/min. More details have been described elsewhere, e.g., [21]. To ensure the correct placement of the probes in the different tissue layers, ultrasound imaging was performed to analyze probe depths in the tissue. After OFM sampling had ended, ultrasound gel was applied to the skin, and measurements were performed horizontally along the open mesh exchange area of the OFM probes. Probe depths were determined retrospectively based on the image sequences using the evaluation software provided by GE Healthcare.

### 2.5. Ex Vivo Experiment

In order to exclusively evaluate diffusive drug delivery without the influence of systemic transport via blood circulation, lidocaine and diclofenac tissue concentrations were analyzed in explanted porcine tissue. Tissue was explanted from the back of two domestic pigs (two samples per animal, left and right of the spine, *n* = 4) at the Division of Biomedical Research at the Medical University of Graz, Austria. Tissue explants consisted of dermal tissue, SAT, and muscle tissue. Tissue explants were delivered to the laboratories of Joanneum Research HEALTH within 90 min after explantation. OFM sampling in tissue explants was performed in a climate chamber with feedback-controlled temperature (32 ± 0.8 °C) and relative humidity (49 ± 4%). Temperature and relative humidity were logged every 10 min with a data logger. After the skin was cleaned, four application sites were demarcated for testing SP-103 5.4%, and two application sites were designated for the application of Pennsaid^®^ 2%. After tissue explants were acclimated for 60 min, two OFM probes per application site were implanted side-by-side in each tissue layer (dermis, SAT, muscle; *n* = 6/application site). SP-103 5.4% topical patches were applied on eight application sites (20 cm^2^ per patch), and Pennsaid^®^ 2% was deposited with a positive displacement pipette over an area of 20 cm^2^ at 15 mg/cm^2^ at four application sites (Table 1). OFM sampling was initiated immediately after product application with a flow rate of 0.5 µL/min (*t* = 0 h). SP-103 5.4% was removed after 12 h. OFM sampling continued until *t* = 24 h, pooling samples every two hours from side-by-side probes in the same tissue. OFM samples were stored immediately at −80 °C and later analyzed using HPLC-MS/MS. The lower limit of quantification (LLOQ) was 2.1 nM for lidocaine and 1.7 nM for diclofenac.

### 2.6. In Vivo Experiment

Animal experiments were conducted at the Division of Biomedical Research at the Medical University of Graz, Austria. The experimental protocols were approved by the Animal Testing Commission of the Federal Government of the Austrian Federal Ministry of Education, Science and Research (#2020-0.547.803). The study was performed on two separate days in four anesthetized female domestic pigs. On average (±SD), pigs were 11.8 ± 1.0 weeks old and weighed 38.3 ± 5.3 kg. Per pig, one application site on each side of the animal’s back was assigned to test items SP-103 5.4%, ZTlido^®^ 1.8%, and Pennsaid^®^ 2% (*n* = 6 sites of 20 cm^2^ each). The induction of anesthesia was conducted after sufficient preoxygenation with propofol 1% (3 mg/kg/kg bolus). For anesthesia, a premedication mixture of midazolam (0.5 mg/kg), azaperone (2 mg/kg), and ketamine (10 mg/kg) was administered intramuscularly. Anesthesia was maintained using propofol 1% (2–5 mg/kg/h) and fentanyl (20 µg/kg/h) given intravenously and—if necessary—sevoflurane gas 1–2%. Furthermore, an isotonic electrolyte solution was administered intravenously at a rate of approximately 10 mL/kg/h during the first hour of anesthesia and 3 mL/kg/h after that. At the end of the OFM sampling, the anesthetized animals were euthanized by intravenous potassium chloride (>2 mmol/kg).

**Figure 2:**
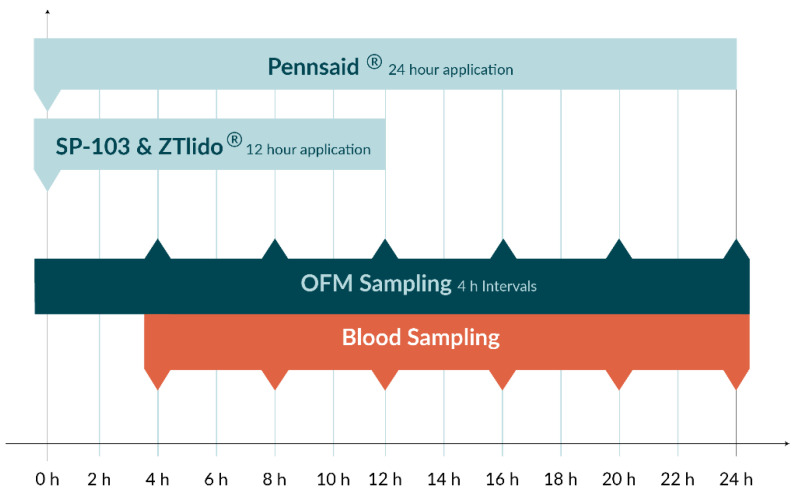
Application and sampling schedule for the in vivo experiment.

After anesthesia was induced, OFM probes were implanted like in the ex vivo experiment with two OFM probes per application site in each tissue layer. At *t* = 0 h, SP-103 5.4% and ZTlido^®^ 1.8% were applied and Pennsaid^®^ 2% was distributed at 15 mg/cm^2^ (Figure 2). OFM sampling was initiated at a flow rate of 0.5 µL/min, and samples were collected every 4 h and pooled from side-by-side probes from the same tissue layer and stored at −80 °C until analysis. Concurrently, blood samples were obtained starting at *t* = 4 h (Figure 2), spun to serum (clot time 30 min, 5 min centrifugation at 10,000× *g* and room temperature), and stored at −80 °C until further analysis. At *t* = 12 h, SP-103 5.4% and ZTlido^®^ 1.8% were removed, and sampling was continued until *t* = 24 h. Concentrations of lidocaine and diclofenac in the OFM and serum samples were subsequently analyzed by HPLC-MS/MS with the same LLOQ as the ex vivo experiment.

### 2.7. HPLC-MS/MS

All samples were extracted by using solid-phase extraction (SPE). After washing and equilibration using acetonitrile and MilliQ water, samples were loaded onto the SPE material. After a wash step using MilliQ water, the samples were eluted with acetonitrile, evaporated under compressed air, and reconstituted in MilliQ water. The method utilized an isocratic elution system comprising 50/50 MilliQ water/acetonitrile. While the injection volume was 6 µL, the column compartment was maintained at 35 °C to enhance chromatographic stability. The typical parameters of the mass spectrometer were as follows: gas temperature 120 °C, gas flow 11 L/min, nebulizer 40 psi, sheath gas temperature 250 °C, sheath gas flow (L/min) 12, capillary voltage (V) 2000, nozzle voltage (V) 2000, funnel high-pressure RF (V) 90, funnel low-pressure RF (V) 100. 13C6 isotopic labeled diclofenac and d10 lidocaine were used as internal standards. The following MRM transitions were used for detection in negative ionization mode: diclofenac 294.0 > 250.0, 13C6-diclofenac 300.0 > 256.0, lidocaine 235.1 > 86.2, and d10-lidocaine 245.1 > 96.2.

### 2.8. Statistics

The area under the drug concentration curve (AUC) was calculated using the trapezoidal method with 4 h intervals (*t* = 4 h, 8 h, 12 h, 16 h, 20 h, 24 h) [22]. If an interval was missing, linear interpolation was applied. If the last interval was missing, the second to last interval served as the final interval. AUC differences were tested for significance using a Wilcoxon rank sum test, and *p* values < 0.05 were considered statistically significant. R software v4.1.1 (https://www.r-project.org, accessed on 10 August 2021) was used for AUC calculations and statistical analyses. All results are presented as mean ± standard deviation, unless stated otherwise.

## 3. Results and Discussion

### 3.1. Diclofenac and Lidocaine Pharmacokinetics (Ex Vivo)

**Figure 3:**
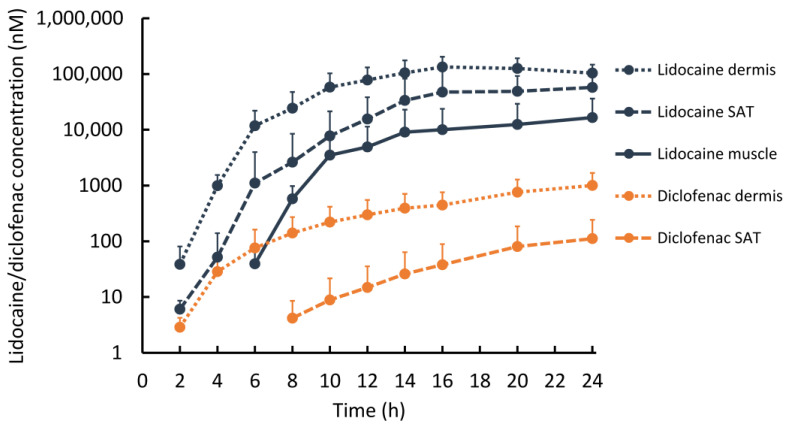
Ex vivo results showing arithmetic mean (+standard deviation) for lidocaine (dark blue, SP-103 5.4%) and diclofenac (orange, Pennsaid^®^ 2%) concentrations (above LLOQ) in the dermis, subcutaneous adipose tissue (SAT), and muscle tissue in porcine tissue explants over 24 h (SP-103 5.4% was removed after 12 h) on a logarithmic scale. Data points represent the preceding 2 h OFM sampling interval. *n* = 8 for lidocaine and *n* = 2 for diclofenac in each tissue layer.

Lidocaine (SP-103 5.4%) and diclofenac (Pennsaid^®^ 2%) concentrations across all tissue layers increased over the first 16 h before reaching a plateau (Figure 3). At 2 h, lidocaine concentrations in the dermis were about 13-fold higher than diclofenac concentrations and about 100-fold higher at the end of the experiment (*p* > 0.05). Tissues in close proximity to the TDS (i.e., dermis) showed a higher concentration of lidocaine and diclofenac compared to deeper tissues (i.e., SAT, muscle). Lidocaine was detected at 2 h in the dermis (38.6 ± 42.2 nM) and SAT (6.1 ± 2.5 nM) and at 6 h in muscle tissue (39.3 ± 29.1 nM). Diclofenac was detected at t = 2 h in the dermis (2.9 ± 1.4 nM) and at *t* = 8 h in SAT (4.2 ± 4.3 nM) but was not detected in muscle tissue throughout the experiment with all concentrations below the LLOQ. The observed difference in the pharmacokinetics of lidocaine and diclofenac can be attributed to the amount of the drug applied (15 mg vs. 6 mg), to the different formulations, and to the protein binding properties of the two drugs. Diclofenac is transported highly efficiently via the blood capillaries because of its high albumin binding (>99%) while lidocaine shows moderate binding affinity to alpha-1-acid glycoprotein, which is present in low concentrations in both the dermis and plasma [23]. Our results indicate that diclofenac did not reach muscle tissue via diffusion in a detectable concentration as the ex vivo experiment had no blood circulation in the explanted tissue and thus exclusively assessed diffusive transport. The ex vivo experiment served the sole purpose of showing that the analgesics can reach deeper tissue layers by diffusion and do not depend on systemic distribution.

### 3.2. Diclofenac and Lidocaine Pharmacokinetics (In Vivo)

**Figure 4:**
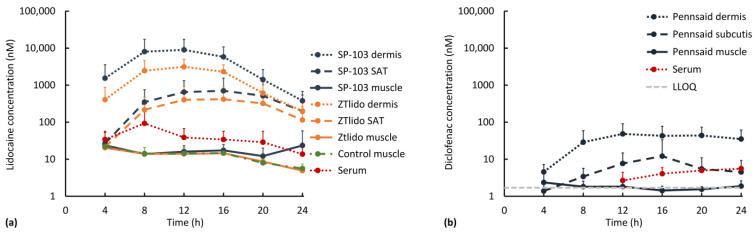
Results from in vivo experiment: (**a**) Arithmetic mean (+standard deviation) of lidocaine concentrations over time in tissues below the application sites for SP-103 5.4% (dark blue) and ZTlido^®^ 1.8% (orange) in dermis (dotted), SAT (dashed), and muscle tissue (solid) shown on a logarithmic scale. The green dashed curve represents concentrations in control muscle tissue where diclofenac was topically applied. *n* = 8 for lidocaine in each tissue layer and *n* = 4 for serum. (**b**) Arithmetic mean (+standard deviation) of diclofenac concentrations over time in tissues below the Pennsaid^®^ 2% application sites in dermal tissue (dotted), SAT (dashed), and muscle tissue (solid) shown on a logarithmic scale. *n* = 8 for diclofenac in each tissue layer and *n* = 4 for serum.

OFM probe depth (mean ± standard deviation) was 1.5 ± 0.3 mm (*n* = 48) in the dermis, 3.3 ± 1.0 mm (*n =* 47) in the SAT, and 8.6 ± 1.7 mm (*n =* 47) in muscle tissue. Probe depths in each tissue layer were thus considered uniform. Overall, and as expected, drug concentrations in all tissues were lower in the in vivo experiment compared to the ex vivo experiment due to systemic redistribution, which was confirmed by detectable concentrations of lidocaine and diclofenac in the serum (Figure 4). In the tissue, peak lidocaine (from both SP-103 5.4% and ZTlido^®^ 1.8%) and diclofenac (from Pennsaid^®^ 2%) concentrations were reached before 24 h, and drug levels decreased towards the end of the experiment (Figure 4). Four hours after application, lidocaine was detected in serum (34.1 ± 22.7 nM) and in all tissue layers at all application sites, with the highest concentration in the dermis (SP-103 5.4%: 1535.5 ± 2032.3 nM and ZTlido^®^ 1.8%: 406.9 ± 465.3 nM; Figure 4a). After the maximum concentration was reached in muscle tissue, lidocaine concentrations decreased in serum (red line), in the control muscle (green line), and at the ZTlido^®^ 1.8% application sites (yellow line), but did not decrease at the SP-103 5.4% sites (blue line), even surpassing serum levels at 24 h (Figure 4a). At 24 h, the lidocaine concentration in muscle tissue was almost 5-fold higher at the SP-103 5.4% site (23.4 ± 34.7 nM) than the ZTlido^®^ 1.8% site (4.9 ± 1.4 nM), and higher than serum (13.7 ± 13.4 nM, 1.7-fold difference). SP-103 5.4% was thus the only test item with a significant amount of diffusion into muscle tissue. Our results are in line with a study published in 2021 that measured lidocaine concentrations for 12 h after a 5% lidocaine patch application using microdialysis and found that lidocaine appeared within one hour in SAT but had a clear delay in plasma samples [24]. Another study successfully detected lidocaine after the use of over-the-counter topical drugs [25], but no study has yet assessed the deep penetration of lidocaine into muscle tissue.

After the application of Pennsaid^®^ 2%, the diclofenac concentration increased first in the dermis and subsequently in SAT (Figure 4b). In the serum, the diclofenac concentration rose above the LLOQ after 12 h, while concentrations in the muscle remained around the LLOQ over 24 h. These in vivo findings are in line with the ex vivo finding that the applied diclofenac concentration was insufficient to penetrate beyond the SAT via diffusion. The exact mechanism of diclofenac diffusion from the dermis into muscle is not fully understood, but factors such as lipophilicity, concentration gradient, low molecular weight, and the presence of blood vessels facilitate its transport into deeper tissue layers [19,26]. One would assume that in the in vivo experiment, systemic diclofenac absorption would lead to an increase in muscle levels due to transport via the bloodstream, which was not the case in this study. This may be explained by the fact that the topical diclofenac formulation is optimized to enhance local effects while minimizing systemic exposure [27]. Therefore, even if some diclofenac molecules penetrate through the dermis, the amount absorbed into the bloodstream is limited. Another possible explanation may be that diclofenac was metabolized almost entirely in the liver [28]. It can be speculated that proper application of the topical diclofenac gel or cream with a proper drug concentration is essential for maximizing its effects. If the gel is not applied directly to the desired muscle area or if it is not adequately massaged into the skin, the diffusion of diclofenac into the muscle may be limited.

**Figure 5:**
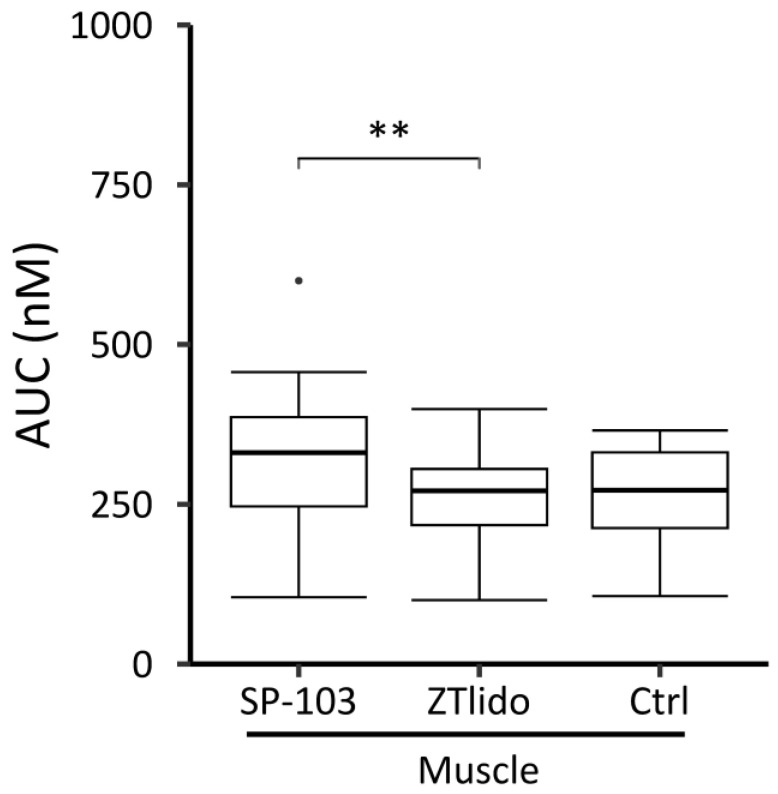
Area under the lidocaine concentration curve (AUC) in muscle tissue over 24 h following application of lidocaine-containing adhesive TDS (SP-103 5.4%, ZTlido^®^ 1.8%) versus control (diclofenac-containing gel TDS Pennsaid^®^, no lidocaine) in the in vivo experiment. Box plots show median with interquartile range and dot indicates an outlier (** *p* < 0.01), *n* = 8.

As diclofenac was not detected in muscle tissue, AUC analysis was performed for lidocaine only. The lidocaine AUC over 24 h in muscle tissue was significantly higher at SP-103 5.4% sites than at ZTlido^®^ 1.8% sites (*p* = 0.008, Figure 5). The median ZTlido^®^ 1.8% AUC (271.2 nM [217.5, 305.5]) was similar to the control AUC (272.0 nM [212.8, 331.3]); however, the difference between the control AUC (no lidocaine application) and SP-103 5.4% AUC (330.9 nM [246.8, 386.6]) was not statistically significant (*p* = 0.078, Figure 5).

A difference in muscle lidocaine concentration was not observed between ZTlido^®^ 1.8% sites (i.e., sites with lower lidocaine TDS concentration) and areas without a lidocaine TDS (ctrl, Figure 5). ZTlido^®^ 1.8% and SP-103 5.4% adhesive formulations are identical except for drug load (i.e., SP-103 5.4% has a 3-fold higher drug load per cm^2^), which resulted in significant differences in lidocaine concentrations in the underlying muscle tissue. 

DThe diffusion of lidocaine occurs along a concentration gradient to areas of low concentration in muscle tissue where lidocaine blocks sodium channels in nerve cell membranes, inducing the anesthetizing and therefore pain-relieving effect [29,30]. It has been shown that local delivery of lidocaine can decrease drug-induced adverse effects substantially and is highly effective in treating pain [31,32,33]. Of note, most published studies have used a 5% lidocaine patch, and there seem to be no other published studies investigating lidocaine pharmacokinetics for adhesive TDS products with a lower drug concentration such as ZTlido^®^ 1.8% approved in the USA in 2018. This may indicate that there is a limited number of lidocaine-delivering TDSs with this level of bioavailability [34]. The results from our in vivo experiment showed that when maintaining the same TDS adhesive formulation but introducing a higher lidocaine drug load, the TDS with the lower drug load (ZTlido^®^ 1.8%) resulted in a systemic lidocaine delivery that surpasses the potential of direct spatial diffusion, delivering drug mainly via blood circulation into the muscle. The 3-fold increase in drug load (SP-103 5.4%) showed that the higher drug concentration delivered to the skin directly above the area of musculoskeletal pain resulted in a higher level of diffusive drug penetrating into the muscle. Based on these data, it is evident that drugs are not only delivered via the systemic pathway but diffusion plays a substantial role in drug delivery when applying adhesive TDSs.

In vivo as well as ex vivo experiments showed the highest drug concentrations in the skin, lower concentrations in the SAT, and the lowest concentrations in the muscle tissue. Overall, lidocaine concentrations were always higher in all tissues compared to diclofenac, and in both experiments, diclofenac showed very low concentrations in the muscle. All in vivo tissue concentrations were lower than ex vivo tissue concentrations except for diclofenac in muscle tissue, where in vivo concentrations were higher than ex vivo concentrations. This indicates that diclofenac is transported into deeper tissue layers to a higher degree via the bloodstream compared to lidocaine. The diclofenac concentration in SAT in the in vivo experiment showed a peak concentration at 16 h, which did not correlate with the peak concentration in the dermis and thus indicates diclofenac clearance via blood circulation. Predominant drug transport via blood circulation is also supported by the finding that in the in vivo experiment, diclofenac concentrations in serum were higher than those in SAT, while for lidocaine, the SAT concentration was approximately 10-fold higher compared to lidocaine serum concentrations. In summary, lidocaine showed a more pronounced transport via local tissue diffusion compared to diclofenac, for which blood transport is the dominant transport route.

### 3.3. Limitations of Ex Vivo and In Vivo Experiments

Our study was limited by several factors: (i) Breathing tubes used during anesthesia were coated in lidocaine, resulting in a baseline serum lidocaine concentration ≠ 0 and probably increased lidocaine concentrations until *t* = 12 h. Since the main finding is after *t* = 12 h in muscle tissue, this limitation did not influence the main outcome of the study. (ii) Due to the limited surface area on the pig’s back, a separate control site with no product application could not be included. Therefore, Pennsaid^®^ 2% sites acted as a negative control for lidocaine relative to SP-103 5.4% and ZTlido^®^ 1.8%. (iii) As this was a small pilot study, the sample size was not sufficient to achieve statistical significance for all tests except for the AUC of lidocaine in muscle tissue.

## 4. Conclusions

To the best of our knowledge, this is the first study to show that analgesic drugs can be effectively delivered into muscle tissue via direct diffusion and systemic drug delivery by using an efficient adhesive TDS. While it is important to note that drug penetration may vary depending on the formulation, application site (size, location), and individual user, the results of this study suggest that topical application of SP-103 5.4% TDS is effective in delivering lidocaine to an area of localized musculoskeletal pain by direct diffusion and could, therefore, be effective in the treatment of musculoskeletal pain disorders.

A combination of an ex vivo setup followed by an in vivo setup also allowed a clear distinction between pure diffusive drug transport (ex vivo) and combination of systemic and diffusive drug transport (in vivo). Compared to standard biopsy studies for TDS research, OFM enables multiple readouts from the exact same tissue and does not require a radiolabeled marker, which can be challenging for TDS manufacturing. OFM was successfully used to monitor drug penetration processes from the dermis via the SAT into muscle tissue and provided a time-resolved concentration profile in these tissue layers. This temporal resolution provides a clear measure for drug onset, lag time between tissues, maximum tissue drug concentration, and the effective time span of an effective drug concentration in each tissue. 

## Figures and Tables

**Table 1:** Summary of test items and test conditions, n.a. = not available.

Product Strength/Concentration	Test Setup	Drug	Drug Load per TDS (mg)	Dimensions of Application Site	Drug Load per Application Site (mg)
SP-103 5.4%	ev vivo/in vivo	lidocaine	108	8 × 2.5 cm	15.4
ZTlido^®^ 1.8%	in vivo	lidocaine	36	8 × 2.5 cm	5.1
Pennsaid^®^ 2%	ev vivo/in vivo	diclofenac	n.a.	8 × 2.5 cm	6.0 (15 mg/cm^2^)

## Data Availability

Data are available to the reader upon reasonable request.
